# Talking about cross-talk: the immune system and the microbiome

**DOI:** 10.1186/s13059-016-0921-4

**Published:** 2016-03-17

**Authors:** David Zeevi, Tal Korem, Eran Segal

**Affiliations:** Department of Computer Science and Applied Mathematics, Weizmann Institute of Science, Rehovot, 7610001 Israel; Department of Molecular Cell Biology, Weizmann Institute of Science, Rehovot, 7610001 Israel

## Abstract

A report on the first EMBO conference entitled “Next Gen Immunology—From Host Genome to the Microbiome: Immunity in the Genomic Era”, held at the Weizmann Institute of Science, Israel, 14–16 February, 2016.

The first Next Gen Immunology EMBO conference, held at the Weizmann Institute of Science in mid-February, touched upon a broad set of topics from host genetics to transcriptomics and microbiome analysis. It specifically demonstrated the intimate cross-talk between the microbiome and the immune system and called for a similarly close interdisciplinary collaboration between microbiologists and immunologists. Recent years have exhibited an increasing number of works on the modulation of the microbiome by the immune system [1–3] and the effect of the microbiome on the healthy development of immunity [4, 5] as well as on autoimmune disorders [6–8]. Groundbreaking research on these topics was presented, along with new research methods, resulting in a broad spectrum of exciting works. Finally, novel therapeutic directions were demonstrated that probe and modulate host–microbiome interactions, and show great promise for future research. Here, we highlight several research topics that are of interest to the scientific and biomedical communities.

## Microbial signatures of disease

With major advances in sequencing came the current advent of microbiome research. In the past two decades the composition and function of the microbiome have been shown to associate with obesity, inflammatory bowel disease, type II diabetes, liver cirrhosis, host nutritional choices, drug treatment, and many other physiological and pathological states. The conference discussed two major drivers in the field of microbiome research: the better and deeper understanding of the microbial signatures themselves and applications of this improved understanding to study finer and more complex host physiological and pathological states.

Quite a few novel approaches for microbiome analysis were presented in the conference. A pioneer in the field of microbiome research, Rob Knight (University of California, San Diego, USA) highlighted the importance of following the microbiome of individuals over time. Using a three-dimensional principal coordinate analysis of the compositions of microbiomes, Knight presented a visual method for microbiome diagnostics. He showed that even though the composition of the microbiome may fluctuate over time, it remains unique for different people. Julie Segre (National Institutes of Health, USA) presented a computational pipeline for the examination of the skin microbiome, allowing the exploration of different strains and of the pangenome versus the core genome of different species. Sharon Greenblum (University of Washington, USA) presented a different pipeline, for the detection of strain-level gene copy number variations.

Many speakers discussed new insights obtained using diverse analysis methods. Knight showed that his visualization method can be used to follow the development of the neonatal microbiome and in the future identify risk for allergies and asthma in aberrantly developing microbiomes. Ramnik Xavier (Broad Institute, USA) examined infants genetically predisposed to type I diabetes and showed that the disease is anticipated by a reduction of diversity in gut microbial species. He further described a difference in bacteria metabolizing human milk oligosaccharide (HMO) in different countries, with a possible contribution to host immunity. A working hypothesis presented was that bacterial colonization, followed by HMO metabolism by immune-silent bacteria, may attenuate immune education and lead to autoimmunity. Moran Yassour (Broad Institute, USA) presented results from a longitudinal cohort of 40 children who consumed or abstained from antibiotic consumption, and showed a prolonged reduction of microbial diversity in children that are prescribed repeated courses of antibiotics, also resulting in less stable communities, reduced intraspecies diversity, and transient increase in antibiotic resistance genes.

Segre showed surprising figures of viral and fungal content in the skin microbiome and a shift in its composition going through puberty. She presented her work on atopic dermatitis, showing that two bacterial species, *Staphylococcus aureus* and *Staphylococcus epidermidis*, rise in abundance during flares of the disease. Eran Elinav (Weizmann Institute of Science, Israel) presented a collaborative project with our lab that addressed the issue of recurrent obesity, which may be linked to the microbiome, thereby impeding dieting and weight management.

## The microbiome as an immunomodulator

Another central theme of the conference was the recognition of the important role of the microbiome in host immune maturation and in immunological health and disease. The microbiome was shown to affect many immune pathways, including innate immunity, inflammasomes, and regulatory T cells.

Andrew MacPherson (University of Bern, Switzerland) showed that the microbiota in pregnancy shapes neonate immunity, and specifically that innate immunity can be transferred between mother and offspring in the absence of the immunomodulating agent. Macpherson colonized pregnant germ-free mice with an *Escherichia coli* strain which infects the host transiently, thus decoupling the effect of gestational and postnatal maternal microbiome. Finally, he showed that such colonization initiates the transfer of microbial molecules to the offspring, causing them to develop innate immunity to the same *E. coli*. The effect of the maternal microbiome on offspring health was underlined by Dan Littman (Skirball Institute of Biomolecular Medicine, New York University, USA) who showed that simulating a viral infection with poly I:C at gestational day 12.5 leads to autism spectrum disorder (ASD)-like symptoms in mice offspring. This process is blocked with anti-interleukin (IL)-6 or anti-IL-17 antibodies which affect maternal T-cell differentiation. Since T-cell differentiation is also modulated by gut microbiota, this work shows a possible role for the maternal microbiome in offspring ASD.

Fiona Powrie (Oxford University, UK) showed that a polysaccharide derived from *Helicobacter hepaticus* regulates IL-10 and T_reg_ response in the gut but not IL-6 and tumor necrosis factor-alpha, modulating the immune response and allowing the bacteria to prosper in the resulting niche. Such discoveries hold promise for future therapies modulating host immunity using microbiome modulation and microbiome-derived metabolites.

## Technological advances

Many new methodological and technological advances were presented in the meeting. Feng Zhang (Massachusetts Institute of Technology, USA) discussed the CRISPR technology, and presented his efforts to improve its specificity by introducing mutations that reduce the ability of Cas9 to stabilize incorrect guide matches. He also presented the process of identifying “new” CRISPR systems, and specifically the FnCpf1 system, which has a simple RNA guide, cuts with sticky end, and is independent of the RNA polymerase III enzyme of the host—allowing for a simpler system which is more efficient in many cases. The potential of the CRISPR technology is just starting to unfold, with works such as that of Jonathan Schmid-Burgk (University of Bonn, Germany) who used it to knockout multiple genes, identifying key genes to NLRP3 inflammasome activation, such as *NEK7*. A complementary approach was taken by Nobel laureate Bruce Beutler (University of Texas Southwestern, USA), who found the dominant role of *NEK7* in the activation of the NLRP3 inflammasome, enforcing mutual exclusivity of cell division and the inflammasome response, by assaying the phenotypic effect in a forward genetic analysis of *N*-ethyl-*N*-nitrosourea-induced mutagenesis in mice.

Timm Schroeder (ETH Zurich, Switzerland) made a claim for continuously following and quantifying the fate of stem cells at the single-cell level. He presented his approach, by combining time-lapse microscopy, cellular markers, and computational analysis. This allows him to identify decision-making points and combine them with single-cell approaches to descend into molecular mechanisms. Ilana Kolodkin-Gal (Weizmann Institute of Science, Israel) utilized X-ray for the study of biofilms, arguing for a dominant role for minerals in their formation and function. She showed that three-dimensional biofilm formation is associated with and dependent on crystalline calcium carbonate deposition, which allows for control of diffusion, potentiating antibiotic resistance within the biofilm.

Michael Fischbach (University of California, San Francisco, USA) presented his methodology for novel biocompound discovery, which involves prediction of molecular products of genetic sequences, instead of the common course of first finding a molecule of interest and then looking for the biosynthesizing enzymes. This is a much needed approach, as even the best-studied organisms have many genes whose products are unknown. The same idea applies to gene clusters in the human microbiome, as most are not characterized, yet appear in over 90 % of the Human Microbiome Project samples. A striking example is indoxyl sulfate, which is created in a manner that is dependent upon microbial metabolism of diet-derived molecules and is toxic to kidney patients.

## Transcription regulation in host immune response

One way by which exogenic compounds exert their effect on the host is by altering transcription, and the topic of transcription regulation was widely discussed during the meeting. Nobel laureate David Baltimore (Caltech, USA) discussed the dynamics of mRNA transcription following inflammation and described how the temporal order of the immune response is regulated by splicing, by measuring the accumulation of unspliced mRNA. Baltimore showed that the splice sites of late-response genes harbor introns that contain conserved noncanonical sequences which serve as “bottlenecks”.

Amos Tanay (Weizmann Institute of Science, Israel) discussed new ways to study topologically associating domains (TADs) and gene regulation within them. Tanay introduced algorithms and new single-cell and single-molecule assays to capture “walks” between physically linked genomic loci. He then argued that multiway contacts between enhancers can be explained by independent pairwise interactions within the context of TADs, while silenced domains can form rosette-like hubs. Ido Amit (Weizmann Institute of Science, Israel) presented a collaboration with Tanay where they examined the transcriptome of single hematopoietic progenitors and used transcriptional sorting to delineate progenitor subgroups, as opposed to contemporary surface markers and functional assays, and found early transcriptional priming of differentiation lines, with no mixed-state progenitors. Furthermore, he demonstrated that comprehensive single-cell analysis of bone marrow progenitors can be used to revise the current hematopoietic tree model, defining new progenitors, markers, signaling pathways, and transcriptional lineage regulators.

## Microbiome as a basis for therapeutics

The microbiome has been implicated as a target for therapy as it affects human health, but, unlike human genetics, it is easier to manipulate. Recurrent *Clostridium difficile* colitis is now commonly treated with stool transplants, and the effect of microbial transplants on obesity and insulin sensitivity has also been shown. However, although probiotic and prebiotic supplementation, along with stool transplants and diet-based microbiome alterations, are some of the tools readily available in the clinic, little is known about the mechanisms leading to their desired effects. Host immunity, the presence or absence of certain metabolites, bacterial composition and bacterial genetics are all important players in the complex environment of the gut, and were all addressed at the conference.

Addressing the effect of host immunity, Richard Flavell (Yale University, USA) observed the fraction of the gut microbiome that is coated by immunoglobulin A (IgA), postulating that it acts as an initiator of immune responses. In a healthy gut, IgA normally coats pathogens, but individuals with inflammatory bowel diseases exhibit aberrant IgA coating of microbes. Flavell introduced the fraction of microbes coated with IgA to germ-free mice, showing their ability to induce dysbiosis and colitis. Finally, as a prospective therapeutic avenue, Flavell showed that immunizing against one of the aberrantly coated species was enough to protect mice against dextran sulfate sodium (DSS)-induced colitis.

The importance of certain metabolites for the healthy function of the gut microbiome was shown by Powrie. Tying metabolite composition with host immunity, she discussed how the short-chain fatty acid butyrate promotes bone marrow-derived macrophage activity with improved Salmonella killing. She ended with a therapeutic promise, showing that treating mice with butyrate promoted host defense against *Citrobacter rodentium* infection. Using an analysis of the compositions of microbiomes, Knight showed that applying a vaginal swab to neonates born by cesarean section promoted a healthier development of infant microbiomes. In patients with a *Clostridium difficile* infection, Knight showed that stool samples cluster outside the normal range of healthy human stool microbiota composition, but that a stool transplant can induce a favorable change within a day. We (presented by Eran Segal) showed a collaborative project with Elinav in which we measured the composition and genetic function of the microbiomes of over 800 people and found them to be associated with an effect on human glucose metabolism. We further showed that these microbial markers are utilized in the prediction of personal response to meals, and that the microbiome changes in a manner that was consistent across different individuals when the host is put on a diet designed to lower postmeal blood glucose responses. This intricate interplay between host diet, host health, and microbiome compositional dynamics holds promise for the design of new diet-based microbiome modulating therapeutics.

## Concluding remarks

This meeting covered many new and exciting developments in microbiome research, immunology, and the cross-talk between them. Due to the large number of exciting and interesting talks presented in the meeting, this report only provides a small glimpse of these topics rather than a comprehensive view of the entire conference. Many unpublished works were presented and we can expect some innovative research to be published very soon.

## Abbreviations

ASD: Autism spectrum disorder
DSS: Dextran sulfate sodium
HMO: Human milk oligosaccharide
IgA: Immunoglobulin A
IL: Interleukin
TAD: Topologically associating domain
